# "Hot cores" in proteins: Comparative analysis of the apolar contact area in structures from hyper/thermophilic and mesophilic organisms

**DOI:** 10.1186/1472-6807-8-14

**Published:** 2008-02-29

**Authors:** Alessandro Paiardini, Riccardo Sali, Francesco Bossa, Stefano Pascarella

**Affiliations:** 1 Dipartimento di Scienze Biochimiche "A. Rossi Fanelli", Università La Sapienza, P.le A. Moro 5, 00185 Roma, Italy

## Abstract

**Background:**

A wide variety of stabilizing factors have been invoked so far to elucidate the structural basis of protein thermostability. These include, amongst the others, a higher number of ion-pairs interactions and hydrogen bonds, together with a better packing of hydrophobic residues. It has been frequently observed that packing of hydrophobic side chains is improved in hyperthermophilic proteins, when compared to their mesophilic counterparts. In this work, protein crystal structures from hyper/thermophilic organisms and their mesophilic homologs have been compared, in order to quantify the difference of apolar contact area and to assess the role played by the hydrophobic contacts in the stabilization of the protein core, at high temperatures.

**Results:**

The construction of two datasets was carried out so as to satisfy several restrictive criteria, such as minimum redundancy, resolution and *R*-value thresholds and lack of any structural defect in the collected structures. This approach allowed to quantify with relatively high precision the apolar contact area between interacting residues, reducing the uncertainty due to the position of atoms in the crystal structures, the redundancy of data and the size of the dataset. To identify the common core regions of these proteins, the study was focused on segments that conserve a similar main chain conformation in the structures analyzed, excluding the intervening regions whose structure differs markedly. The results indicated that hyperthermophilic proteins underwent a significant increase of the hydrophobic contact area contributed by those residues composing the alpha-helices of the structurally conserved regions.

**Conclusion:**

This study indicates the decreased flexibility of alpha-helices in proteins core as a major factor contributing to the enhanced termostability of a number of hyperthermophilic proteins. This effect, in turn, may be due to an increased number of buried methyl groups in the protein core and/or a better packing of alpha-helices with the rest of the structure, caused by the presence of hydrophobic beta-branched side chains.

## Background

Earth's environments exhibit the most diverse physico-chemical conditions, including extremes of temperature, pressure, salinity and pH. Among these factors, temperature certainly exerts a deep selective pressure on cell biochemistry and physiology [1]. Indeed, temperatures approaching 100°C usually denature proteins and nucleic acids, and increase the fluidity of membranes to lethal levels [2]. It is therefore of great interest to study how organisms coped with the molecular adaptations required to thrive in extreme environments, particularly at high temperatures. Such organisms, which are distributed among the three domains of life, are called "thermophiles" or "hyperthermophiles", if they exhibit an optimal growth in either a 45°C – 80°C or a 80°C – 110°C temperature range, respectively [3].

To date, a number of studies has been carried out to understand how proteins found in hyper/thermophilic organisms are stabilized [1-6]. Thanks to the wealth of sequence and structural information available today on hyper/thermophilic proteins, it is becoming clear that there is not a general rule for the stabilization of proteins at high temperatures. Rather, an increased thermal stability seems to be achieved through a combination of different small structural modifications involving, amongst the others, ion-pairs interactions, hydrogen bonds and packing of hydrophobic residues [6].

Regarding the latter, one frequently invoked theory is that the packing of hydrophobic side chains is improved in thermophilic and hyperthermophilic proteins, when compared to their mesophilic counterparts [7]. Many studies on proteins adaptation to high temperatures focused on the differences in compactness between hyper/thermophilic and mesophilic proteins using accessible surface area [6] or cavity size [8] as judgment criteria. However, as discussed by Robinson-Rechavi and Godzik [9], and by Gromiha [10], these approaches present several drawbacks, e.g., the individual contribution to the enhanced thermostability of different structural environments and inter-residue contacts cannot be assessed. Hence, alternative ways to quantify protein compactness were adopted. For example, Gromiha [10] analyzed the long range and inter-residue contacts in mesophilic and thermophilic proteins of sixteen different protein families, and found that an increase in contacts between hydrogen-bond forming residues increases protein stability. Very recently, the contact order [11] is receiving increasing attention, thanks to the findings obtained by Godzik and his research group [9,12], who found that hyperthermophilic proteins from *T. maritima *have higher contact order than their mesophilic counterparts. Most importantly, contact order is correlated to the folding rate of proteins that fold with a two-states mechanism [11].

**Figure 1:**
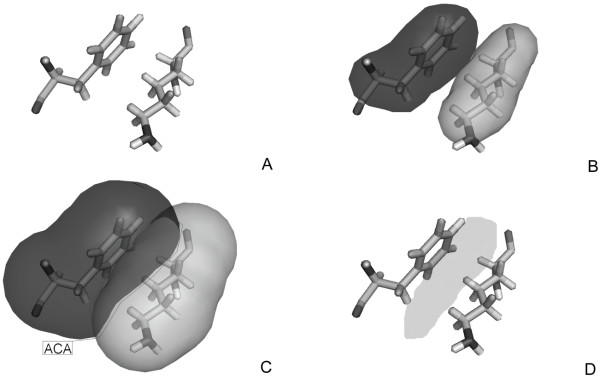
**Computation of the apolar contact area**. A-B) Initially, for each amino acid pair (in this case two sample residues, Phe and Lys, are considered), the Van der Walls surface is generated. C) Then, the solvent accessible surface is computed. D) The latter is used to compute the hydrophobic contact surface between the two interacting residues.

However, a severe limitation of this and other [10,13] studies is that two residues are considered to be in contact if the distance between their C_α _atoms or between one atom and any other atom is below an arbitrary threshold. For example, Robinson-Rechavi *et al*. [12] considered two residues to be in contact if any of their atoms are closer than 4.5 Å, while Gromiha [10] made use of a sphere of 8.0 Å centered on C_α _atoms to define long-range contacts. Furthermore, this approach bears another important drawback: it does not permit to quantify the hydrophobic contact area between two interacting residues. The hydrophobic contact area between buried residues represents in fact an indirect measure of both entropic (entropy change due to the rearrangement of the local water molecules as two hydrophobic residues interact [14]) and enthalpic (van der Waals forces in protein core, due to tight packing of neighboring residues [4]) effects (Figure 1).

Therefore, despite a series of experimental and theoretical studies on the molecular mechanisms of protein folding [15,16] and stability [3,9,17] argued that the hydrophobic contacts play a role of paramount importance in such processes, the difference of apolar contact area between large datasets of proteins from hyper/thermophilic organisms and their mesophilic homologs, to our knowledge, has been never quantified.

Such consideration, along with the wealth of information provided very recently by structural genomics projects, prompted the comparison of a large number of protein crystal structures from hyper/thermophilic organisms and their mesophilic homologs, in order to assess the role played by the hydrophobic contacts in the stabilization of the protein core, at high temperatures.

## Results

### Analysis of the Apolar Contact Area

**Figure 2:**
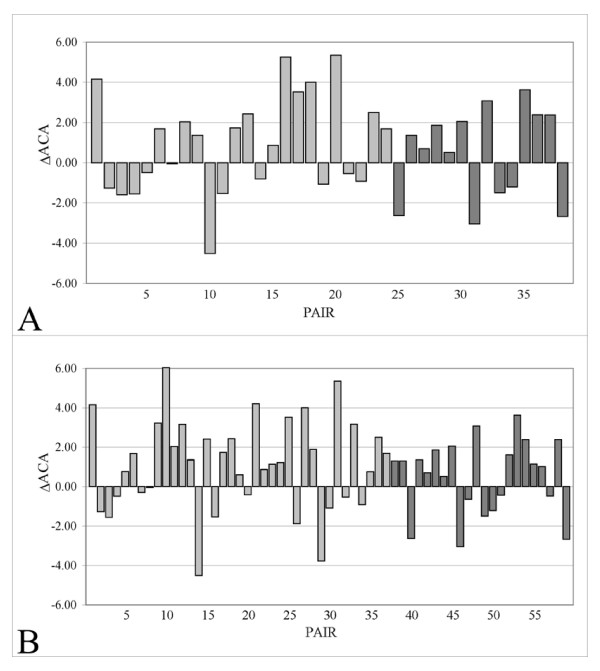
**Differences in the apolar contact area (ΔACA) for each protein pair, composing dataset A and B, computed over the whole protein structure**. Values for hyperthermophilic/mesophilic protein pairs and thermophilic/mesophilic pairs are expressed in Å^2^/residue and represented as light grey and dark grey bars, respectively. Numbers on X-axis refer to Table 1 (A) and Table 2 (B).

Two datasets were obtained from a collection of 1563 hyperthermophilic and thermophilic proteins, retrieved from structural databases using several keywords (see Methods section; Table 1 and 2). In the first case a choice criteria favouring quality over quantity of data yielded a non redundant dataset, which will be referred to as "*A*", including 38 crystal structures, lacking any structural defect and displaying a maximum resolution of 2.0 Å and a maximum *R-value *of 0.25. Dataset A represents a subset of a second dataset, which will be referred to as "*B*". Dataset B is composed of 59 crystal structures lacking any structural defect, displaying a maximum resolution of 3.0 Å and a maximum *R-value *of 0.30. For each structure composing the two datasets, a mesophilic homologous counterpart was collected, following the same above mentioned choice criteria. The computation of the total apolar contact area (*ACA*) between the residues of each structure pair composing dataset *A *and *B *was then carried out. The statistical significance of the observed differences of *ACA *between hyper/thermophilic proteins and their mesophilic counterparts was assessed with a paired *t*-test. The results are reported in Table 3 (see also Additional file 1 for additional information). T-test values are expressed as the associated probability *P *of acceptance of the null hypothesis, that is, there are no significant differences of *ACA *between hyper/thermophilic and mesophilic pairs. T-values scoring > 2.0 (P(*t*) < 0.05) are considered statistically significant. Figure 2 shows the difference of apolar contact area computed over the whole structures of the protein pairs composing the two analysed datasets. The obtained values were normalized by the sequence length of each protein. In dataset *A*, 22 (13 hyperthermophilic/mesophilic and 9 thermophilic/mesophilic protein pairs) of the 38 considered protein pairs showed an increase of the *ACA *(Figure 2A); the corresponding P(*t*) was ~0.086 (0.079 for hyperthermophiles and 0.690 for thermophiles). In dataset *B*, 38 (24 hyperthermophilic/mesophilic and 14 thermophilic/mesophilic protein pairs) of the 59 protein pairs showed an increase of the *ACA *(Figure 2B); the corresponding P(*t*) was ~0.012 (0.020 for hyperthermophiles and 0.474 for thermophiles). Although the obtained differences were not considered statistically significant, according to the t-test validation analysis, for both datasets (Table 3), nonetheless they indicated a general increase of the apolar contact area in hyperthermophilic proteins, compared to their mesophilic counterparts.

**Table 1:** Hyperthermophilic/Mesophilic (1–24) and Thermophilic/Mesophilic (25–38) pairs in dataset A*

ID	PDB	Class	Organism	Res (Å)	PDB	Class	Mesophile	Res (Å)	ΔÅ	%identity	Functional Class	Description
1	1A2Z A	a/b	*Thermococcus litoralis*	1.73	1AUG A	a/b	*Bacillus amyloliquefaciens*	2.00	0.27	37	Peptidase	Pyrrolidone Carboxyl Peptidase
2	1A53 0	a/b	*Sulfolobus solfataricus*	2.00	1PII 0	a/b	*Escherichia coli*	2.00	0.00	38	Synthase	Indole-3-Glycerolphosphate Synthase
3	1DD3 A	a/b	*Thermotoga maritima*	2.00	1CTF 0	a/b	*Escherichia coli*	1.70	0.3	69	Ribosomal	Ribosomal Protein
4	1DQI A	mainly b	*Pyrococcus furiosus*	1.70	1DFX 0	mainly b	*D. desulfuricans*	1.90	0.20	34	Oxidoreductase	Superoxide Reductase
5	1FTR A	a+b	*Methanopyrus kandleri*	1.70	1M5S A	a+b	*Methanosarcina barkeri*	1.85	0.15	59	Transferase	Formyltransferase
6	1G29 1	a/b	*Thermococcus litoralis*	1.90	1B0U A	a/b	*Salmonella typhimurium*	1.50	0.40	31	Sugar Binding	Malk Protein
7	1HQK A	a/b	*Aquifex aeolicus*	1.60	1W19 A	a/b	*M. tuberculosis*	2.00	0.40	50	Transferase	Lumazine Synthase
8	1IU8 A	a/b	*Pyrococcus horikoshii*	1.60	1AUG A	a/b	*Bacillus amyloliquefaciens*	2.00	0.40	45	Hydrolase	Pyrrolidone-Carboxylate Peptidase
9	1J31 A	a/b	*Pyrococcus horikoshii*	1.60	1UF5 A	a/b	*Agrobacterium sp*.	1.60	0.00	31	Unknown	Hypothetical Protein Ph0642
10	1JI0 A	a/b	*Thermotoga maritima*	2.00	1G6H A	a/b	*Escherichia coli*	1.60	0.40	31	Carrier	Abc Transporter
11	1JVB A	a/b	*Sulfolobus solfataricus*	1.85	1M6H A	a/b	*Homo sapiens*	2.00	0.15	31	Oxidoreductase	Alcohol Dehydrogenase
12	1LK5 A	a/b	*Pyrococcus horikoshii*	1.75	1M0S A	a/b	*Haemophilus influenzae*	1.90	0.15	42	Isomerase	D-Ribose-5-Phosphate Isomerase
13	1M2K A	a/b	*Archaeoglobus fulgidus*	1.47	1S5P A	a/b	*Escherichia coli*	1.96	0.49	41	Trascriptional Regulator	Sir2 Homologue
14	1M5H A	a+b	*Archaeoglobus fulgidus*	2.00	1M5S A	a+b	*Methanosarcina barkeri*	1.85	0.15	68	Transferase	Formyltransferase
15	1NSJ 0	a/b	*Thermotoga maritima*	2.00	1PII 0	a/b	*Escherichia coli*	2.00	0.00	33	Isomerase	P-Ribosylanthranilate Isomerase
16	1P1L A	a/b	*Archaeoglobus fulgidus*	2.00	1NAQ A	a/b	*Escherichia coli*	1.70	0.3	33	Unknown	Cation Resistent Protein Cut-A
17	1U1I A	a/b	*Archaeoglobus fulgidus*	1.90	1P1J A	a/b	*Saccharomyces cerevisiae*	1.70	0.20	31	Isomerase	Myo-Inositol Phosphate Synthase
18	1UKU A	a/b	*Pyrococcus horikoshii*	1.45	1NAQ A	a/b	*Escherichia coli*	1.70	0.25	39	Metal Binding Protein	Cation Resistent Protein Cut-A
19	1V3W A	mainly b	*Pyrococcus horikoshii*	1.50	1XHD A	mainly b	*Bacillus cereus*	1.90	0.40	40	Lyase	Ferripyochelin Binding Protein
20	1V7R A	a/b	*Pyrococcus horikoshii*	1.40	1K7K A	a/b	*Escherichia coli*	1.50	0.10	34	Hydrolase	Hypothetical Protein Ph1917
21	1VE0 A	a/b	*Sulfolobus tokodaii*	2.00	1VMH A	a/b	*C. acetobutylicum*	1.31	0.69	42	Metal Binding Protein	Hypothetical Protein St2072
22	1VPE 0	a/b	*Thermotoga maritima*	2.00	1HDI A	a/b	*Sus scrofa*	1.80	0.20	47	Transferase	Phosphoglycerate Kinase
23	1XGS A	mainly a	*Pyrococcus furiosus*	1.75	1B6A 0	mainly a	*Homo sapiens*	1.60	0.15	40	Aminopeptidase	Methionine Aminopeptidase
24	1XTY A	a/b	*Pyrococcus abyssi*	1.80	1Q7S A	a/b	*Homo sapiens*	2.00	0.20	48	Hydrolase	Peptidyl-Trna Hydrolase
25	1EE8 A	mainly a	*Thermus thermophilus*	1.90	1TDZ A	mainly a	*Lactococcus lactis*	1.80	0.10	35	Dna Binding Protein	Fpg Protein
26	1GD7 A	mainly b	*Thermus thermophilus*	2.00	1PXF A	mainly b	*Escherichia coli*	1.87	0.13	34	Rna Binding Protein	Csaa Protein
27	1J09 A	a/b	*Thermus thermophilus*	1.80	1NZJ A	a/b	*Escherichia coli*	1.50	0.30	33	Ligase	Glutamil-Trna Synthase
28	1J3N A	a/b	*Thermus thermophilus*	2.00	1E5M A	a/b	*Synechocystis sp*.	1.54	0.46	55	Transferase	Acyl Carrier Protein
29	1JBO A	mainly a	*T. elongatus*	1.45	1B8D A	mainly a	*Griffithsia monilis*	1.90	0.45	38	Photosynthesis	Phycocyanin
30	1MNG A	mainly a	*Thermus thermophilus*	1.80	1GV3 A	mainly a	*Anabaena sp*.	2.00	0.20	59	Oxidoreductase	Superoxide Dismutase
31	1SRV A	a/b	*Thermus thermophilus*	1.70	1KID 0	a/b	*Escherichia coli*	1.70	0.00	69	Chaperone	Groel
32	1UZB A	a/b	*Thermus thermophilus*	1.40	1O0A A	a/b	*Halobacterium salinarum*	1.42	0.02	34	Oxidoreductase	1-Pyrroline-5-Carboxylate Dehydrogenase
33	1V6S A	a/b	*Thermus thermophilus*	1.50	16PK 0	a/b	*Trypanosoma brucei*	1.60	0.10	43	Transferase	Phosphoglycerate Kinase
34	1V8F A	a/b	*Thermus thermophilus*	1.90	1N2E A	a/b	*M. tuberculosis*	1.60	0.30	55	Ligase	Pantothenate Synthetase
35	1VC4 A	a/b	*Thermus thermophilus*	1.80	1PII 0	a/b	*Escherichia coli*	2.00	0.20	37	Lyase	Indole-3-Glycerolphosphate Synthase
36	1VCD A	a/b	*Thermus thermophilus*	1.70	1SJY A	a/b	*Deinococcus radiodurans*	1.39	0.31	34	Hydrolase	Ap6a Hydroxylase Ndx1
37	1YYA A	a/b	*Thermus thermophilus*	1.60	1MO0 A	a/b	*Caenorhabditis elegans*	1.70	0.10	44	Isomerase	Triosephosphate Isomerase
38	2PRD 0	a/b	*Thermus thermophilus*	2.00	1SXV A	a/b	*M. tuberculosis*	1.30	0.70	51	Hydrolase	Inorganic Pyrophosphatase

* Optimal growth temperatures are between 50°C and 80°C for thermophiles, and above 80°C for hyperthermophiles

**Table 2:** Hyperthermophilic/Mesophilic (1–38) and Thermophilic/Mesophilic (39–59) pairs in dataset B

ID	PDB	Class	Organism	Res (Å)	PDB	Class	Mesophile	Res (Å)	ΔÅ	%identity	Functional Class	Description
1	1A2Z A	a/b	*Thermococcus litoralis*	1.73	1AUG A	a/b	*Bacillus amyloliquefaciens*	2.00	0.27	37	Peptidase	Pyrrolidone Carboxyl Peptidase
2	1A53 0	a/b	*Sulfolobus solfataricus*	2.00	1PII 0	a/b	*Escherichia coli*	2.00	0.00	38	Synthase	Indole-3-Glycerolphosphate Synthase
3	1DQI A	mainly b	*Pyrococcus furiosus*	1.70	1DFX 0	mainly b	*Desulfovibrio desulfuricans*	1.90	0.20	34	Oxidoreductase	Superoxide Reductase
4	1FTR A	a+b	*Methanopyrus kandleri*	1.70	1M5S A	a+b	*Methanosarcina barkeri*	1.85	0.15	59	Transferase	Formyltransferase
5	1DD3 A	a/b	*Thermotoga maritima*	2.00	1CTF 0	a/b	*Escherichia coli*	1.70	0.3	69	Ribosomal	Ribosomal Protein
6	1G29 1	a/b	*Thermococcus litoralis*	1.90	1B0U A	a/b	*Salmonella typhimurium*	1.50	0.40	31	Sugar Binding	Malk Protein
7	1HDG O	a/b	*Thermotoga maritima*	2.50	1RM4 A	a/b	*Spinacia oleracea*	2.00	0.50	56	Oxidoreductase	Glyceraldehyde 3 Phosphate Dehydrogenase
8	1HQK A	a/b	*Aquifex aeolicus*	1.60	1W19 A	a/b	*Mycobacterium tuberculosis*	2.00	0.40	50	Transferase	Lumazine Synthase
9	1I4N A	a/b	*Thermotoga maritima*	2.50	1PII 0	a/b	*Escherichia coli*	2.00	0.50	34	Lyase	Indole-3-Glycerolphosphate Synthase
10	1IOF A	a/b	*Pyrococcus furiosus*	2.20	1AUG A	a/b	*Bacillus amyloliquefaciens*	2.00	0.20	43	Hydrolase	Pyrrolidone-Carboxylate Peptidase
11	1IU8 A	a/b	*Pyrococcus horikoshii*	1.60	1AUG A	a/b	*Bacillus amyloliquefaciens*	2.00	0.40	45	Hydrolase	Pyrrolidone-Carboxylate Peptidase
12	1J0A A	a/b	*Pyrococcus horikoshii*	2.50	1TZJ A	a/b	*Pseudomonas sp*.	1.99	0.51	31	Lyase	Aminocyclopropane Carboxylate Deaminase
13	1J31 A	a/b	*Pyrococcus horikoshii*	1.60	1UF5 A	a/b	*Agrobacterium sp*.	1.60	0.00	31	Unknown	Hypothetical Protein Ph0642
14	1JI0 A	a/b	*Thermotoga maritima*	2.00	1G6H A	a/b	*Escherichia coli*	1.60	0.40	31	Carrier	Abc Transporter
15	1JJI A	a/b	*Archaeoglobus fulgidus*	2.20	1JKM B	a/b	*Bacillus subtilis*	1.85	0.35	35	Hydrolase	Carboxylesterase
16	1JVB A	a/b	*Sulfolobus solfataricus*	1.85	1M6H A	a/b	*Homo sapiens*	2.00	0.15	31	Oxidoreductase	Alcohol Dehydrogenase
17	1LK5 A	a/b	*Pyrococcus horikoshii*	1.75	1M0S A	a/b	*Haemophilus influenzae*	1.90	0.15	42	Isomerase	D-Ribose-5-Phosphate Isomerase
18	1M2K A	a/b	*Archaeoglobus fulgidus*	1.47	1S5P A	a/b	*Escherichia coli*	1.96	0.49	41	Trascriptional Regulator	Sir2 Homologue
19	1M4Y A	a+b	*Thermotoga maritima*	2.10	1G3K A	a+b	*Haemophilus influenzae*	1.90	0.20	66	Hydrolase	Hslv
20	1M5H A	a+b	*Archaeoglobus fulgidus*	2.00	1M5S A	a+b	*Methanosarcina barkeri*	1.85	0.15	68	Transferase	Formyltransferase
21	1MXG A	a/b	*Pyrococcus woesei*	1.60	1VJS 0	a/b	*Bacillus licheniformis*	1.70	0.10	31	Idrolasi	AAmilase
22	1NSJ 0	a/b	*Thermotoga maritima*	2.00	1PII 0	a/b	*Escherichia coli*	2.00	0.00	33	Isomerase	P-Ribosylanthranilate Isomerase
23	1P1L A	a/b	*Archaeoglobus fulgidus*	2.00	1NAQ A	a/b	*Escherichia coli*	1.70	0.3	33	Unknown	Cation Resistent Protein Cut-A
24	1OJU A	a/b	*Archaeoglobus fulgidus*	2.79	1GUZ A	a/b	*Chlorobium vibrioforme*	2.00	0.79	34	Oxidoreductase	Malate Dehydrogenase
25	1U1I A	a/b	*Archaeoglobus fulgidus*	1.90	1P1J A	a/b	*Saccharomyces cerevisiae*	1.70	0.20	31	Isomerase	Myo-Inositol Phosphate Synthase
26	1UE8 A	mainly a	*Sulfolobus tokodaii*	3.00	1ODO A	mainly a	*Streptomyces coelicolor*	1.85	1.15	32	Unknown	Cytochrome P450
27	1UKU A	a+b	*Pyrococcus horikoshii*	1.45	1NAQ A	a+b	*Escherichia coli*	1.70	0.25	39	Metal Binding Protein	Cation Resistent Protein Cut-A
28	1ULZ A	a/b	*Aquifex aeolicus*	2.20	1DV1 A	a/b	*Escherichia coli*	1.90	0.30	53	Ligase	Pyruvate Carboxylase
29	1UVV A	a/b	*Thermotoga maritima*	2.75	1GS5 A	a/b	*Escherichia coli*	1.50	1.25	35	Transferase	Acetylglutamate Kinase
30	1V3W A	mainly b	*Pyrococcus horikoshii*	1.50	1XHD A	mainly b	*Bacillus cereus*	1.90	0.40	40	Lyase	Ferripyochelin Binding Protein
31	1V7R A	a/b	*Pyrococcus horikoshii*	1.40	1K7K A	a/b	*Escherichia coli*	1.50	0.10	34	Hydrolase	Hypothetical Protein Ph1917
32	1VE0 A	a/b	*Sulfolobus tokodaii*	2.00	1VMH A	a/b	*Clostridium acetobutylicum*	1.31	0.69	42	Metal Binding Protein	Hypothetical Protein St2072
33	1VFF A	a/b	*Pyrococcus horikoshii*	2.55	1E4I A	a/b	*Bacillus polymyxa*	2.00	0.55	32	Hydrolase	B-Glucosidase
34	1VPE 0	a/b	*Thermotoga maritima*	2.00	1HDI A	a/b	*Sus scrofa*	1.80	0.20	48	Transferase	Phosphoglycerate Kinase
35	1WPW A	a/b	*Sulfolobus tokodaii*	2.80	1A05 A	a/b	*Thiobacillus ferrooxidans*	2.00	0.80	40	Oxidoreductase	Ipm Dehydrogenase
36	1XGS A	mainly a	*Pyrococcus furiosus*	1.75	1B6A 0	mainly a	*Homo sapiens*	1.60	0.15	39	Aminopeptidase	Methionine Aminopeptidase
37	1XTY A	a/b	*Pyrococcus abyssi*	1.80	1Q7S A	a/b	*Homo sapiens*	2.00	0.20	48	Hydrolase	Peptidyl-Trna Hydrolase
38	1B33 A	mainly a	*M. laminosus*	2.30	1XG0 C	mainly a	*Rhodomonas*	0.97	1.33	32	Photosynthesis	Allophycocianin
39	1BXB A	a/b	*Thermus aquaticus*	2.20	1MUW A	a/b	*Streptomyces olivochromogenes*	0.86	1.34	58	Isomerase	Xilose Isomerase
40	1EE8 A	mainly a	*Thermus thermophilus*	1.90	1TDZ A	mainly a	*Lactococcus lactis*	1.80	0.10	35	Dna Binding Protein	Fpg Protein
41	1GD7 A	mainly b	*Thermus thermophilus*	2.00	1PXF A	mainly b	*Escherichia coli*	1.87	0.13	34	Rna Binding Protein	Csaa Protein
42	1J09 A	a/b	*Thermus thermophilus*	1.80	1NZJ A	a/b	*Escherichia coli*	1.50	0.30	33	Ligase	Glutamil-Trna Synthase
43	1J3N A	a/b	*Thermus thermophilus*	2.00	1E5M A	a/b	*Synechocystis sp*.	1.54	0.46	55	Transferase	Acyl Carrier Protein
44	1JBO A	mainly a	*T. elongatus*	1.45	1B8D A	mainly a	*Griffithsia monilis*	1.90	0.45	38	Photosynthesis	Phycocyanin
45	1MNG A	mainly a	*Thermus thermophilus*	1.80	1GV3 A	mainly a	*Anabaena sp*.	2.00	0.20	59	Oxidoreductase	Superoxide Dismutase
46	1SRV A	a/b	*Thermus thermophilus*	1.70	1KID 0	a/b	*Escherichia coli*	1.70	0.00	69	Chaperone	Groel
47	1UKW A	mainly a	*Thermus thermophilus*	2.40	1RX0 A	mainly a	*Homo sapiens*	1.77	0.63	39	Oxidoreductase	Acil-Coa Dehydrogenase
48	1UZB A	a/b	*Thermus thermophilus*	1.40	1O0A A	a/b	*Halobacterium salinarum*	1.42	0.02	34	Oxidoreductase	1-Pyrroline-5-Carboxylate Dehydrogenase
49	1V6S A	a/b	*Thermus thermophilus*	1.50	16PK 0	a/b	*Trypanosoma brucei*	1.60	0.10	44	Transferase	Phosphoglycerate Kinase
50	1V8F A	a/b	*Thermus thermophilus*	1.90	1N2E A	a/b	*Mycobacterium tuberculosis*	1.60	0.30	55	Ligase	Pantothenate Synthetase
51	1V8G A	a/b	*Thermus thermophilus*	2.10	1VQU A	a/b	*Nostoc sp*.	1.85	0.25	42	Transferase	Anthranilate Phosphoribosyltransferase
52	1VC2 A	a/b	*Thermus thermophilus*	2.60	1GAD O	a/b	*Escherichia coli*	1.80	0.80	51	Oxidoreductase	Glyceraldehyde 3 Phosphate Dehydrogenase
53	1VC4 A	a/b	*Thermus thermophilus*	1.80	1PII 0	a/b	*Escherichia coli*	2.00	0.20	37	Lyase	Indole-3-Glycerolphosphate Synthase
54	1VCD A	a/b	*Thermus thermophilus*	1.70	1SJY A	a/b	*Deinococcus radiodurans*	1.39	0.31	34	Hydrolase	Ap6a Hydroxylase Ndx1
55	1WXD A	a/b	*Thermus thermophilus*	2.10	1NYT A	a/b	*Escherichia coli*	1.50	0.60	36	Oxidoreductase	Shikimate 5-Dehydrogenase
56	1XAA 0	a/b	*Thermus thermophilus*	2.10	1CNZ A	a/b	*Salmonella typhimurium*	1.76	0.34	52	Oxidoreductase	3-Isopropylmalate Dehydrogenase
57	1YYA A	mainly b	*Thermus thermophilus*	1.60	1MO0 A	mainly b	*Caenorhabditis elegans*	1.70	0.10	44	Isomerase	Triosephosphate Isomerase
58	1YKF A	a/b	*T. brockii*	2.50	1JQB A	a/b	*Clostridium beijerinckii*	0.53	1.97	77	Oxidoreductase	Nadp-Dependent Alcohol Dehydrogenase
59	2PRD 0	a/b	*Thermus thermophilus*	2.00	1SXV A	a/b	*Mycobacterium tuberculosis*	1.30	0.70	52	Hydrolase	Inorganic Pyrophosphatase

**Table 3:** T-tests results for the *ACA *distributions, measured in different structural environments*

	**ACA Distributions^+^**			
	**Structural environment**			
*P *≤ 0.05**	**Total**	**SCRs**	**α-Helices in SCRs**	**β-strands in SCRs**

**All**				
Dataset A	0.0864	0.0640	0.0859	0.9437
Dataset B	**0.0124**	**0.0069**	**0.0159**	0.1745
Shapiro-Wilk Test°	0.90/0.99	0.07/0.002°°	0.96/0.59	
**Hyperthermophiles**				
Dataset A	0.0790	**0.0029**	**0.0524**	0.8120
Shapiro-Wilk Test°		0.26/0.90	0.97/0.16	
Dataset B	**0.0205**	**0.0001**	**0.0113**	0.061
Shapiro-Wilk Test°	0.53/0.42	0.49/0.36	0.13/0.003°°°	
**Thermophiles**				
Dataset A	0.6901	0.5139	0.8387	0.7080
Dataset B	0.3357	0.7530	0.3123	0.6027

* Values are expressed as the associated probability *P *of acceptance of the null hypothesis

** *P *≤ 0.05 are considered statistically significant, and are bolded

+ The statistical significance of the observed differences of ACA between hyper/thermophilic proteins and their mesophilic counterparts

°The obtained *P*(*t*) of the Shapiro-Wilk test for significant results. The distributions of *ACA *are presented in the form hyper/thermophilic-mesophilic distribution

°°The obtained *P*(*t*) of the Shapiro-Wilk test is 0.46 removing 2 outliers; P(t) of the associated t-test = 0.005 removing the outliers

°°°The obtained *P*(*t*) of the Shapiro-Wilk test is 0.62 removing 3 outliers; P(t) of the associated t-test = 0.001 removing the outliers

**Figure 3:**
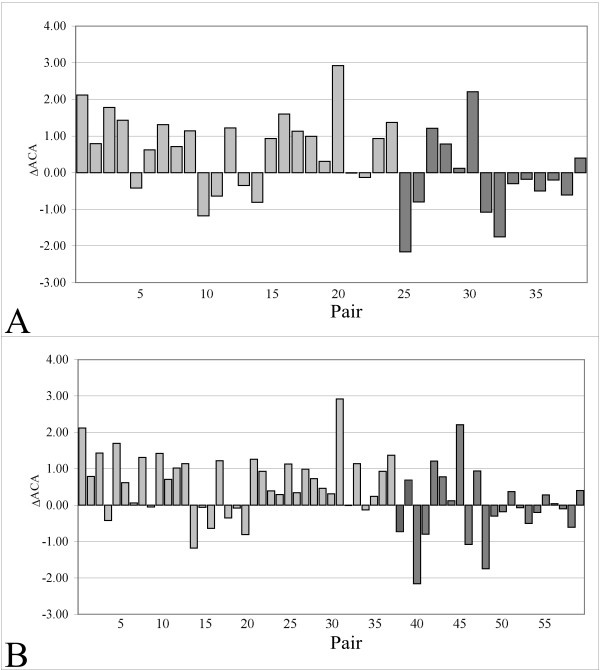
**Differences in the apolar contact area (ΔACA) for each protein pair, composing dataset A and B, computed over the SCRs**. Values for hyperthermophilic/mesophilic protein pairs and thermophilic/mesophilic pairs are expressed in Å^2^/residue and represented as light grey and dark grey bars, respectively. Numbers on X-axis refer to Table 1 (A) and Table 2 (B).

A more detailed analysis on the structurally conserved regions [18] (SCRs; see methods section) of the structures composing dataset *A *and *B *indicated that, in both datasets, a number of hyperthermophilic proteins underwent a highly significant (*P*(*t*) < 0.001) increase of the hydrophobic contact area of those residues composing the SCRs (Figure 3; Table 3). SCRs were defined as regions displaying a similar local conformation, lacking insertions and deletions and composed of at least three consecutive residues. SCRs are therefore protein segments that conserve the same main-chain conformation in each pair of structures analysed, excluding the intervening regions whose structure differs markedly amongst different proteins [19]. Considering the role of great importance played by the hydrophobic contacts in stabilizing and possibly driving the protein folding mechanism, it seemed interesting to analyse how, during evolution, the SCRs coped with the modifications of the hydrophobic contacts necessary to achieve the correct fold at high temperatures. In dataset *A *(Figure 3A), 22 (17 hyperthermophilic/mesophilic and 5 thermophilic/mesophilic protein pairs, respectively) of the 38 considered protein pairs showed an increase of the *ACA *(P(*t*) ~0.0029). The same trend was also observed for dataset *B *(Figure 3B), in which 37 of 59 protein pairs (27 hyperthermophilic/mesophilic and 10 thermophilic/mesophilic) displayed an increased *ACA *in the direction mesophile → hyper/thermophile (P(*t*) ~0.0001). The measured mean Δ*ACA *was 0.39 Å^2^/residue and 0.37 Å^2^/residue for datasets *A *and *B*, respectively. However, if only the hyperthermophilic/mesophilic pairs were considered, the mean Δ*ACA *was 0.74 Å^2^/residue and 0.63 Å^2^/residue for datasets *A *and *B*, respectively. The maximum measured difference was 2.92 Å^2^/residue for the pair 1V7R/1K7K (nucleotide triphosphate pyrophosphatase from *P. horikoshii/E. coli*). Since these quite high differences of *ACA *can be due to other factors than acquired thermostability (i.e., different overall conformations), the t-test validation analysis was repeated without these extreme pairs, obtaining again not significant results (see "Methods" section and supplementary material).

**Figure 4:**
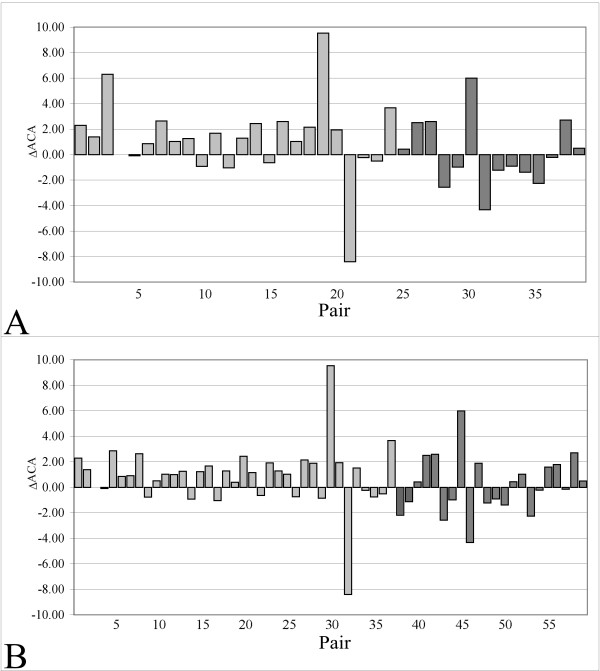
**Differences in the apolar contact area (ΔACA) for each protein pair, composing dataset A and B, computed over the α-helices of the SCRs**. Values for hyperthermophilic/mesophilic protein pairs and thermophilic/mesophilic pairs are expressed in Å^2^/residue and represented as light grey and dark grey bars, respectively. Numbers on X-axis refer to Table 1 (A) and Table 2 (B).

To get a deeper insight into the statistically significant increase of the hydrophobic contact area of protein cores from hyperthermophilic organisms, the possible occurrence of a larger amount of hydrophobic contact area has been examined in different secondary structure elements. In dataset *A *(Figure 4A), 16 out of the 24 hyperthermophilic proteins considered showed an increase of *ACA *in the α-helices of the protein core, compared to their mesophilic counterparts, while in dataset *B *(Figure 4B) the same ratio was 25 out of 37 proteins, with a measured significance P(*t*) ~0.0524 and P(*t*) ~0.0113 for datasets *A *and *B*, respectively. Although in this latter case significant deviations from normality, as judged by the application of the Shapiro-Wilk normality test, were observed for the distribution of mesophilic values, nonetheless removing three outliers gave a Shapiro-Wilk *P*(t) ~0.62 and a t-test *P*(t) ~0.001. These results indicated that α-helices are mainly involved in the increased amount of hydrophobic contact area which was observed comparing hyperthermophilic/mesophilic proteins. Conversely, no statistically significant trends have been observed in the comparison of the *ACA *in the β-strands of the SCRs (Table 3). In dataset *A*, 21 (14 hyperthermophilic/mesophilic protein pairs) of the 38 considered protein pairs showed an increase of the *ACA*, while in dataset *B*, 34 (24 hyperthermophilic/mesophilic proteins) of the 59 pairs exhibited an increase of the *ACA*. The mean value of Δ*ACA *is -0.02 Å^2^/residue and 0.34 Å^2^/residue for dataset *A *and *B*. Therefore, at least for the hyperthermophilic/mesophilic protein pairs, it can be concluded that the statistically significant increase of the hydrophobic contact area of protein cores involves mainly the α-helices and not the β-strands.

### Differences in the amino acid composition of the residues involved in conserved hydrophobic contacts

The differences of amino acid composition of the residues involved in conserved hydrophobic contacts (CHCs; Table 4) [19] between hyperthermophilic proteins and their mesophilic counterparts is expressed in units of standard deviation from the measured mean value, *R*^*aa*^. *R*^*aa *^values > 0 or < 0 indicate, respectively, a frequency of residue type *aa *higher or lower than the expected mean. *R*^*aa *^values ≥ 3.0 standard deviations (*P *≤ 0.01) from the mean value (that approximates zero) were considered statistically significant. Compositional analysis shows no statistically significant differences between hyperthermophilic and mesophilic proteins, regarding the identity of the residues involved in the formation of hydrophobic contacts, except for isoleucine, that scored at ~3.6 standard deviations from the mean in both datasets *A *and *B*. It is important to emphasize that, in evaluating the differences of amino acid composition of the residues involved in conserved hydrophobic contacts, dataset *B*, containing 13 hyperthermophilic/mesophilic protein pairs more than dataset *A*, is probably more confident. In any case, since both datasets *A *and *B *gave very similar results, the role played by isoleucine is probably independent from the number and type of structures analysed.

**Table 4:** Amino acid composition of CHCs*

**DATASET A**		**DATASET B**	
**Amino acid**	**Hyperthermophiles vs. Mesophiles**	**Amino acid**	**Hyperthermophiles vs. Mesophiles**
**A**	-1.045	**A**	-0.680
**V**	-0.107	**V**	-0.115
**F**	0.305	**F**	0.216
**I**	**3.661**	**I**	**3.635**
**L**	-1.609	**L**	-1.585
**D**	-0.451	**D**	-0.365
**E**	0.211	**E**	0.432
**G**	-0.058	**G**	-0.136
**K**	0.130	**K**	0.645
**S**	-0.245	**S**	-0.355
**T**	-0.398	**T**	-0.554
**Y**	0.471	**Y**	0.821
**C**	-0.850	**C**	-0.683
**N**	0.285	**N**	0.231
**Q**	-0.813	**Q**	-0.933
**P**	0.334	**P**	0.207
**M**	-0.036	**M**	-0.412
**R**	0.500	**R**	0.114
**H**	-0.167	**H**	-0.284
**W**	-0.407	**W**	-0.398

* Values are expressed in units of standard deviation from the mean (Z-score). *R *values ≥ 3.0 are considered statistically significant and are bolded.

### Preferred amino acid interactions in conserved hydrophobic contacts

In order to further investigate the statistically significant increase of isoleucine in CHCs of hyperthermophilic proteins, compared to their mesophilic counterparts, an analysis was carried out to infer which amino acid pairs are preferred in the formation of hydrophobic contacts. Preferred amino acid pairs forming hydrophobic contacts were identified by computing the number of times a particular pair of residues comprised in SCRs makes a hydrophobic contact, displaying an apolar contact area > 0.0 Å^2^. The results of this analysis are shown in Tables 5 and 6, where each element *ij *of the interaction matrix reports, in units of standard deviation from the mean value, the measured frequency of interaction between residue *i *and residue *j*. For dataset *A*, accounting for 17864 apolar contacts, five types of interactions (Ile/Ala, Ile/Val, Ile/Phe, Ile/Ile and Ile/Leu) showed a frequency ≥ 3.0 standard deviations from the mean value; in every case, isoleucine is involved in such interactions. Similar results were obtained for dataset *B*, where 33546 interactions were counted: of six types of interactions scoring at > 3.0 standard deviations, five (Ile/Ala, Ile/Val, Ile/Tyr, Ile/Ile and Ile/Leu) involved the amino acid isoleucine. The other statistically significant interaction is between glutamate and lysine, scoring at 3.28 standard deviations from the mean. The closeness between the apolar atoms composing Glu and Lys residues might be only a secondary effect in the generation of strong ion-pairs between these two residues.

**Table 5:** Preferred amino acid interactions in CHCs. Hyperthermophilic versus mesophilic proteins of dataset A are compared*

	ALA	VAL	PHE	ILE	LEU	ASP	GLU	GLY	LYS	SER	THR	TYR	CYS	ASN	GLN	PRO	MET	ARG	HIS	TRP	XXX
ALA	-2.03																				
VAL	-0.36	-0.55																			
PHE	0.85	-0.46	-0.26																		
ILE	**3.07**	**6.09**	**3.17**	**4.33**																	
LEU	-4.00	-1.11	0.42	**3.82**	-4.56																
ASP	-1.23	0.05	-0.49	0.88	-0.23	-0.13															
GLU	-0.83	0.46	0.49	0.04	2.71	-0.13	0.95														
GLY	-0.60	-0.52	-0.35	0.81	-0.45	-0.51	0.92	0.01													
LYS	-1.73	-1.03	0.46	2.45	1.13	-0.48	2.37	0.39	0.98												
SER	-0.87	-0.35	-0.08	1.13	-0.60	-0.16	0.07	-0.81	0.48	0.11											
THR	-1.48	-0.43	-0.40	0.02	-1.03	0.04	-1.01	0.52	0.29	0.13	0.16										
TYR	-0.23	1.54	0.17	1.44	-1.51	0.37	-0.19	0.22	-0.49	0.38	-0.08	0.53									
CYS	-1.76	-1.79	-0.67	-0.57	-3.03	-0.17	-0.01	-0.59	-0.35	-0.77	-0.37	-0.30	-0.12								
ASN	0.67	0.20	0.01	0.33	-0.82	-0.27	0.22	0.24	0.16	-0.23	-0.56	-0.59	0.06	0.06							
GLN	-1.19	-0.88	0.12	0.23	-2.61	-0.34	-1.13	-0.55	0.31	-0.56	-1.32	-0.56	-0.16	-0.04	-0.23						
PRO	0.03	-0.73	0.36	0.27	0.10	0.15	0.71	0.75	0.49	-0.16	-0.32	-0.21	-0.48	0.44	-0.49	0.21					
MET	-0.85	0.44	-0.08	1.22	-0.23	-0.29	0.79	-0.61	0.31	0.23	0.29	-0.25	-0.57	0.15	-0.23	-0.12	0.04				
ARG	-0.31	1.08	0.15	1.65	1.01	-0.12	1.51	-0.04	0.10	-0.07	-0.05	0.49	0.05	-0.18	-0.60	0.34	0.43	0.49			
HIS	-0.50	0.23	-0.13	0.28	-1.55	-0.24	-0.92	0.06	-0.93	-0.11	-0.24	0.16	-0.55	-0.05	-0.02	-0.02	-0.05	-0.14	0.37		
TRP	0.01	-0.40	-0.19	0.64	0.55	0.20	0.95	-0.11	-0.25	-0.08	-0.06	-0.30	-0.14	-0.48	-0.04	0.25	-0.23	0.46	-0.48	0.21	
XXX	0.35	0.09	0.39	0.61	0.74	0.22	0.35	0.04	0.09	0.23	0.26	0.23	0.00	0.05	0.09	0.18	0.00	0.06	-0.08	0.09	0.13

* Values are expressed in units of standard deviation from the mean (Z-score). Values ≥ 3.0 are considered statistically significant and are bolded. Mean = 0.00; standard deviation = 0.10.

**Table 6:** Preferred amino acid interactions in CHCs. Thermophilic versus mesophilic proteins of dataset B are compared*

	ALA	VAL	PHE	ILE	LEU	ASP	GLU	GLY	LYS	SER	THR	TYR	CYS	ASN	GLN	PRO	MET	ARG	HIS	TRP	XXX
ALA	-0.81																				
VAL	-0.80	-0.62																			
PHE	0.29	-0.96	-0.09																		
ILE	**3.27**	**6.36**	2.80	**4.21**																	
LEU	-1.86	-2.02	0.68	**4.17**	-4.10																
ASP	-0.76	-0.23	-0.58	1.21	-0.49	-0.23															
GLU	0.13	0.58	0.51	0.79	1.37	0.11	0.89														
GLY	-0.44	-0.77	-0.50	1.11	-0.26	-0.53	0.57	-0.34													
LYS	-0.46	0.10	0.75	2.51	1.69	0.37	**3.28**	0.65	1.16												
SER	-1.04	-1.38	-0.36	1.47	-0.05	-0.22	0.05	-0.63	0.78	0.06											
THR	-2.05	-1.15	-0.80	0.17	-0.90	-0.12	-0.89	0.00	0.49	-0.15	0.42										
TYR	0.60	1.74	0.90	**3.06**	-0.54	0.67	0.64	0.49	0.84	0.53	0.48	0.86									
CYS	-1.56	-1.49	-0.83	-0.64	-2.55	-0.14	-0.08	-0.48	-0.26	-0.57	-0.53	-0.30	-0.12								
ASN	0.48	0.31	0.15	-0.02	-0.70	-0.04	0.23	0.35	0.49	-0.11	-0.42	-0.15	0.01	0.08							
GLN	-1.58	-1.09	-0.33	-0.38	-2.48	-0.88	-1.02	-0.79	-0.27	-0.73	-1.02	-0.65	-0.24	-0.19	-0.42						
PRO	0.13	-0.94	0.47	0.16	-0.37	0.04	0.72	0.62	0.70	-0.22	-0.26	-0.01	-0.47	0.21	-0.75	0.09					
MET	-0.86	-0.50	0.09	1.19	-0.84	-0.21	0.72	-0.53	0.35	0.05	0.09	-0.52	-0.60	0.01	-0.36	0.11	-0.05				
ARG	-1.22	0.26	-0.11	1.52	-0.12	-0.44	0.75	0.02	-0.20	-0.61	-0.34	0.68	-0.12	-0.11	-0.71	0.10	0.03	0.24			
HIS	-0.51	0.01	-0.34	0.12	-1.45	-0.25	-0.85	0.15	-0.95	-0.28	-0.31	0.20	-0.47	-0.03	-0.26	0.11	-0.05	-0.43	0.14		
TRP	-0.01	-0.23	-0.42	0.58	0.30	0.04	0.77	-0.29	0.03	-0.05	-0.15	-0.09	-0.32	-0.28	-0.09	0.19	-0.17	0.33	-0.55	0.32	
XXX	0.27	0.01	0.28	0.37	0.51	0.17	0.24	0.00	0.08	0.15	0.25	0.17	0.00	0.03	0.06	0.15	0.00	0.03	-0.07	0.09	0.10

* Values are expressed in units of standard deviation from the mean (Z-score). Values ≥ 3.0 are considered statistically significant and are bolded. Mean = 0.00; standard deviation = 0.12.

### Preferred amino acid substitutions in conserved hydrophobic contacts

Favoured amino acid substitutions between the hyperthermophilic and mesophilic proteins were calculated from the results obtained by the CHC_FIND tool [19]. The residues exchange analysis was indeed limited to the identified conserved hydrophobic contacts. The obtained substitution matrices are shown in Tables 7 and 8. Values are expressed in units of standard deviation from the mean. Only values scoring at 3.0 standard deviations or more from the mean were considered statistically significant. Again, almost all of the most significant exchanges involve isoleucine in both datasets (dataset *A*: Val→Ile 6.32, Leu→Ile 6.36; dataset *B*: Val→Ile 6.39, Leu→Ile 6.84 and Phe→Ile 3.12). These exchanges are reflected in the variation of average amino acid composition of hyperthermophiles (Table 4), where a marked increase of isoleucine content can be detected. The only other exchange observed not involving isoleucine is Ala→Val, scoring at 3.20 standard deviations from the mean.

**Table 7:** Preferred amino acid substitutions in CHCs. Hyperthermophilic versus mesophilic proteins of dataset A are compared*

	**TO HYPERTHERMOPHILE**																					
		ALA	VAL	PHE	ILE	LEU	ASP	GLU	GLY	LYS	SER	THR	TYR	CYS	ASN	GLN	PRO	MET	ARG	HIS	TRP	XXX
**FROM MESOPHILE**	ALA	0.00	**3.20**	1.28	1.67	-0.85	0.26	1.79	0.47	1.92	-0.13	-2.22	0.04	-2.48	-0.38	0.43	-0.30	-1.02	-0.13	-0.64	0.09	0.00
	VAL	-3.20	0.00	1.07	**6.31**	-1.58	-0.30	0.21	-0.60	0.13	0.21	-1.79	0.38	-2.09	-0.73	-0.13	0.34	0.34	0.90	0.04	-0.26	0.00
	PHE	-1.28	-1.07	0.00	2.31	-0.73	-0.26	0.04	-0.09	0.38	-0.21	0.60	0.51	-0.21	-0.38	-0.30	0.13	1.54	-0.47	-0.21	-0.64	0.00
	ILE	-1.67	-6.31	-2.31	0.00	-6.36	0.47	0.51	-0.60	0.60	-0.21	0.30	-0.09	-0.34	-0.77	-0.90	0.00	0.90	-1.11	-1.02	-0.09	-0.17
	LEU	0.85	1.58	0.73	**6.36**	0.00	0.13	-0.90	0.30	-1.02	-0.30	0.21	-0.13	-1.71	0.26	-0.90	0.13	-1.62	1.37	-0.68	0.38	0.00
	ASP	-0.26	0.30	0.26	-0.47	-0.13	0.00	1.32	0.09	0.73	-0.13	-0.21	0.09	0.00	-0.77	0.09	-0.13	0.00	0.13	0.51	0.04	0.00
	GLU	-1.79	-0.21	-0.04	-0.51	0.90	-1.32	0.00	-0.30	-0.94	-0.73	-0.47	0.04	-0.47	-0.43	-1.07	-0.47	-0.09	0.17	-0.17	-0.17	0.00
	GLY	-0.47	0.60	0.09	0.60	-0.30	-0.09	0.30	0.00	-0.30	-1.28	0.38	0.38	0.00	0.51	-0.51	-0.09	0.00	0.77	0.17	0.00	0.00
	LYS	-1.92	-0.13	-0.38	-0.60	1.02	-0.73	0.94	0.30	0.00	0.00	-1.02	-0.38	-0.30	-0.04	-1.11	-0.26	0.04	0.77	-0.60	0.13	0.00
	SER	0.13	-0.21	0.21	0.21	0.30	0.13	0.73	1.28	0.00	0.00	1.58	0.00	0.09	-0.56	-0.43	0.30	0.13	0.43	-0.09	-0.13	0.00
	THR	2.22	1.79	-0.60	-0.30	-0.21	0.21	0.47	-0.38	1.02	-1.58	0.00	-0.21	-0.51	0.04	0.26	0.34	-0.30	0.34	0.56	-0.47	0.00
	TYR	-0.04	-0.38	-0.51	0.09	0.13	-0.09	-0.04	-0.38	0.38	0.00	0.21	0.00	-0.81	-0.26	-0.13	0.17	0.43	-0.85	0.34	0.43	0.00
	CYS	2.48	2.09	0.21	0.34	1.71	0.00	0.47	0.00	0.30	-0.09	0.51	0.81	0.00	0.13	0.04	0.17	0.90	0.13	0.26	0.00	0.00
	ASN	0.38	0.73	0.38	0.77	-0.26	0.77	0.43	-0.51	0.04	0.56	-0.04	0.26	-0.13	0.00	-0.85	-0.04	-0.26	-0.13	-0.13	0.13	0.00
	GLN	-0.43	0.13	0.30	0.90	0.90	-0.09	1.07	0.51	1.11	0.43	-0.26	0.13	-0.04	0.85	0.00	0.56	-0.38	0.38	0.13	0.43	0.00
	PRO	0.30	-0.34	-0.13	0.00	-0.13	0.13	0.47	0.09	0.26	-0.30	-0.34	-0.17	-0.17	0.04	-0.56	0.00	-0.17	0.17	-0.21	0.09	0.00
	MET	1.02	-0.34	-1.54	-0.90	1.62	0.00	0.09	0.00	-0.04	-0.13	0.30	-0.43	-0.90	0.26	0.38	0.17	0.00	-0.64	-0.47	0.38	-0.30
	ARG	0.13	-0.90	0.47	1.11	-1.37	-0.13	-0.17	-0.77	-0.77	-0.43	-0.34	0.85	-0.13	0.13	-0.38	-0.17	0.64	0.00	-0.98	0.43	0.00
	HIS	0.64	-0.04	0.21	1.02	0.68	-0.51	0.17	-0.17	0.60	0.09	-0.56	-0.34	-0.26	0.13	-0.13	0.21	0.47	0.98	0.00	0.00	0.00
	TRP	-0.09	0.26	0.64	0.09	-0.38	-0.04	0.17	0.00	-0.13	0.13	0.47	-0.43	0.00	-0.13	-0.43	-0.09	-0.38	-0.43	0.00	0.00	0.00
	XXX	0.00	0.00	0.00	0.17	0.00	0.00	0.00	0.00	0.00	0.00	0.00	0.00	0.00	0.00	0.00	0.00	0.30	0.00	0.00	0.00	0.00

* Values are expressed in units of standard deviation from the mean (Z-score). Values ≥ 3.0 are considered statistically significant and are bolded. Mean = 0.00; standard deviation = 23.41.

**Table 8:** Preferred amino acid substitutions in CHCs. Hyperthermophilic versus mesophilic proteins of dataset B are compared*

	**TO HYPERTHERMOPHILE**																					
		ALA	VAL	PHE	ILE	LEU	ASP	GLU	GLY	LYS	SER	THR	TYR	CYS	ASN	GLN	PRO	MET	ARG	HIS	TRP	XXX
**FROM MESOPHILE**	ALA	0.00	1.73	0.66	2.91	-0.76	0.07	1.54	0.14	1.82	-0.26	-2.44	0.59	-2.34	-0.50	0.43	0.69	-0.47	-0.24	-0.80	-0.31	0.00
	VAL	-1.73	0.00	1.47	**6.39**	-0.92	0.17	0.76	-0.59	0.69	0.62	-1.47	0.43	-1.23	-0.52	-0.28	0.12	0.21	0.31	-0.21	-0.90	0.00
	PHE	-0.66	-1.47	0.00	**3.12**	-1.56	-0.26	-0.05	-0.24	0.05	-0.33	-0.14	1.80	-0.14	-0.31	-0.40	0.14	0.59	-0.31	-0.35	-0.83	0.00
	ILE	-2.91	-6.39	-3.12	0.00	-6.84	0.31	0.38	-0.64	0.85	-0.50	-0.38	0.07	-0.38	-0.54	-0.78	-0.35	0.31	-0.88	-0.57	-0.09	-0.09
	LEU	0.76	0.92	1.56	**6.84**	0.00	0.07	-0.31	0.14	1.09	0.17	0.43	1.35	-0.76	-0.17	-1.56	0.21	-2.96	0.40	-0.40	0.64	0.00
	ASP	-0.07	-0.17	0.26	-0.31	-0.07	0.00	0.80	0.17	0.92	-0.24	-0.28	0.21	0.07	0.07	-0.17	-0.05	-0.14	0.02	0.09	0.28	0.00
	GLU	-1.54	-0.76	0.05	-0.38	0.31	-0.80	0.00	-0.50	-0.43	-0.78	-0.33	0.33	-0.14	-0.35	-0.90	-0.59	-0.05	-0.66	-0.52	0.05	0.00
	GLY	-0.14	0.59	0.24	0.64	-0.14	-0.17	0.50	0.00	0.21	-1.02	0.28	0.28	-0.17	0.35	-0.57	-0.21	0.05	0.45	0.26	-0.07	0.00
	LYS	-1.82	-0.69	-0.05	-0.85	-1.09	-0.92	0.43	-0.21	0.00	0.02	-0.73	-0.40	-0.17	-0.14	-1.99	-0.52	0.00	-0.64	-0.66	0.31	0.00
	SER	0.26	-0.62	0.33	0.50	-0.17	0.24	0.78	1.02	-0.02	0.00	0.50	0.14	0.17	0.43	-0.35	0.21	0.00	0.19	-0.07	-0.12	0.00
	THR	2.44	1.47	0.14	0.38	-0.43	0.28	0.33	-0.28	0.73	-0.50	0.00	0.62	-0.62	-0.33	-0.12	-0.09	0.09	0.21	0.21	-0.26	0.00
	TYR	-0.59	-0.43	-1.80	-0.07	-1.35	-0.21	-0.33	-0.28	0.40	-0.14	-0.62	0.00	-0.52	-0.21	-0.24	-0.21	0.00	-1.16	-0.54	0.69	0.00
	CYS	2.34	1.23	0.14	0.38	0.76	-0.07	0.14	0.17	0.17	-0.17	0.62	0.52	0.00	0.19	0.02	0.14	0.57	0.07	0.28	0.19	0.00
	ASN	0.50	0.52	0.31	0.54	0.17	-0.07	0.35	-0.35	0.14	-0.43	0.33	0.21	-0.19	0.00	-0.95	-0.05	-0.19	-0.33	-0.35	-0.02	0.00
	GLN	-0.43	0.28	0.40	0.78	1.56	0.17	0.90	0.57	1.99	0.35	0.12	0.24	-0.02	0.95	0.00	0.21	-0.02	0.57	0.35	0.24	0.00
	PRO	-0.69	-0.12	-0.14	0.35	-0.21	0.05	0.59	0.21	0.52	-0.21	0.09	0.21	-0.14	0.05	-0.21	0.00	-0.50	0.26	-0.17	0.07	0.00
	MET	0.47	-0.21	-0.59	-0.31	2.96	0.14	0.05	-0.05	0.00	0.00	-0.09	0.00	-0.57	0.19	0.02	0.50	0.00	-0.62	-0.21	0.14	-0.17
	ARG	0.24	-0.31	0.31	0.88	-0.40	-0.02	0.66	-0.45	0.64	-0.19	-0.21	1.16	-0.07	0.33	-0.57	-0.26	0.62	0.00	-0.92	0.50	0.00
	HIS	0.80	0.21	0.35	0.57	0.40	-0.09	0.52	-0.26	0.66	0.07	-0.21	0.54	-0.28	0.35	-0.35	0.17	0.21	0.92	0.00	0.38	0.00
	TRP	0.31	0.90	0.83	0.09	-0.64	-0.28	-0.05	0.07	-0.31	0.12	0.26	-0.69	-0.19	0.02	-0.24	-0.07	-0.14	-0.50	-0.38	0.00	0.00
	XXX	0.00	0.00	0.00	0.09	0.00	0.00	0.00	0.00	0.00	0.00	0.00	0.00	0.00	0.00	0.00	0.00	0.17	0.00	0.00	0.00	0.00

* Values are expressed in units of standard deviation from the mean (Z-score). Values ≥ 3.0 are considered statistically significant and are bolded. Mean = 0.00; standard deviation = 42.10

## Discussion

The main goal of this study was to evaluate on a quantitative basis the relationship between hydrophobic contacts and proteins adaptation to high temperatures.

An essential prerequisite to carry out such a study is to assemble a large and minimally redundant set of very high resolution crystal structures. Indeed, despite the observation that each protein family seems to adopt different structural strategies to adapt to high temperatures [5], common trends may be outlined if a large number of structural data is available [8]. At the same time, since computed values of apolar contact area are mostly influenced by the relative position of the interacting residues, their precision is affected by the resolution of the crystal structures analysed. Therefore two datasets were culled from a set of 1563 crystal structures from thermophilic (optimal growth temperature between 50°C and 80°C) and hyperthermophilic (optimal growth temperature above 80°C) organisms, and their mesophilic counterparts. The rationale of this choice was to assure that the obtained results were not biased either by the paucity of data, or by the quality of the collected crystal structures.

As already discussed by Chen *et al*. [7], the increase of the apolar contact area in hyperthermophilic and thermophilic proteins may be achieved at least by two different mechanisms: an evenly distributed increase over all residues; a local increase over key residues. The latter mechanism, that has been shown to be a major contribute to the enhanced thermostability of proteins from *T. maritima *[9], seems to involve mainly residues already implied in the formation of hydrophobic contacts. This suggests that a better compactness may originate from an even better connectivity in those protein regions that already have a tendency to compactness and not by simply "tightening the loops" [9]. The results obtained in this work on the difference of apolar contact area (Δ*ACA*) agree with this hypothesis: a significant increase of *ACA *was measured in both datasets only when the analysis was limited to the SCRs of the hyperthermophilic structures. The SCRs were presumably subject to similar constraints during the divergent evolution of a family of proteins from a common ancestor, and therefore they possibly contain most of the determinants necessary to maintain the fold. Considering the role played by hydrophobic contacts in this sense, it is not surprising that the residues composing the SCRs and engaging hydrophobic contacts were mostly involved in the structural modifications necessary to achieve and maintain a proper fold at high temperatures. Moreover, the finding that the measure of the difference of *ACA *resulted highly significant only when limited to the SCRs, could explain some apparently not significant results previously obtained by measuring accessible surface area [8] or cavity size [6].

The statistically significant increase of ~0.75 Å^2^/residue of apolar contact area was observed only in the SCRs of hyperthermophilic proteins. Therefore, it can be argued that proteins from thermophilic organisms usually adopt different strategies to enhance thermostability. Indeed, it has been demonstrated that moderately and extremely thermostable proteins rely on different mechanisms to achieve greater stability [8,20]. Ion-pairs interactions represent presumably a predominant force in thermophilic proteins, as well as in many hyperthermophilic proteins [8,21]. On the other hand, comparisons of mesophilic and hyperthermophilic protein structures indicate that the hydrophobic effect has a contribution to stability only at high temperatures, while only moderately thermophilic proteins show an increase in the polarity of their exposed surface [20]. Two factors could be responsible for this difference: the temperature dependence of the thermodynamic forces involved in protein stabilization, and/or the phylogenetic origin of the extremely thermophilic organisms, that belong to the domain Archaea, and are therefore distinct from moderately thermophilic organisms, which are mostly Bacteria. In any case, the obtained results strongly suggest that packing of hyperthermophilic proteins, in comparison with their mesophilic homologs, has improved significantly, and it is reasonable to deduce that this increased amount of apolar contact area contributes to the stabilization of the native state of the protein.

Our analysis revealed that α-helices were mainly involved in the increased amount of *ACA*. Surprisingly, no statistically significant trends have been observed in the comparison of the *ACA *in the β-strands of the SCRs. We cannot provide a clear explanation of this different behaviour between secondary structures. An intriguing possibility is that β-strands are, generally, already almost optimally packed, even in mesophilic proteins, resulting in a small margin of improvement. However, it is also possible that this observation is due to 'sample bias' e.g., the peculiarities of the available protein structures.

Structural stabilization of α-helices in protein cores may therefore represent a component of great importance for the enhanced termostability of hyperthermophilic proteins. A number of studies in the past has stressed the importance of the enhanced stability of α-helices as a general feature of many hyperthermophilic proteins. In order to investigate the role of α-helices in protein thermostability, Petukhov *et al*. [22] compared energy characteristics of α-helices from four families of hyperthermophilic and mesophilic proteins, using statistical mechanical theory for describing helix/coil transitions. They found that the magnitude of the observed decrease in intrinsic free energy on α-helix formation of the thermostable proteins was sufficient to explain the experimentally determined increase of their thermostability. Furthermore, protein engineering studies showed that a well-packed α-helix structure is related to large increase in thermostability [23,24]. It is well known that the flexibility of α-helices is often required to assure protein function, such as conformational transitions in substrate binding or protein-protein interactions [25]. However, an excessive flexibility of this secondary structure element, at high temperatures, could result in an insufficient stability to maintain its native conformation, causing the entire protein to unfold.

According to thermodynamic studies on model peptides in aqueous environments, two main factors appear to play a key role in the structural stability of the α-helices: the presence of amino acids with intrinsic helical propensity, and side chain-side chain interactions [26,27]. Therefore, we further investigated the nature of the increased stabilization of α-helices composing the SCRs of hyperthermostable proteins, determining the differences in amino acid composition of the residues involved in CHCs. The results of this analysis strongly suggest that isoleucine and, to a lesser extent valine, mostly to the detriment of leucine, are involved in the formation of more hydrophobic contacts in hyperthermophilic proteins, compared to their mesophilic counterparts. Likewise, the importance of isoleucine in the formation of CHCs of hyperthermophilic proteins was confirmed by the analysis of the preferred amino acid interactions in CHCs, where almost all types of interactions scoring at > 3.0 standard deviations involved the amino acid isoleucine, and by the favoured amino acid substitutions between the hyperthermophilic and mesophilic proteins in CHCs. A large amount of theoretical and experimental studies demonstrates the importance of isoleucine in the stabilization of protein structures from thermophilic organisms. Malakauskas and Mayo [24] reported the computer-aided engineering of a seven-fold mutant of the β1 domain of the Streptococcal protein G, exhibiting a melting temperature above 100°C and an enhancement in thermodynamic stability of 4.3 kcal mol^-1 ^at 50°C over the wild-type protein. Of seven mutations, five were of type XXX→ Ile, and they improved side-chain packing in the interior of the protein. An increased content of isoleucine in thermophilic and hyperthermophilic proteins, to the detriment of leucine, was also noted by Haney *et al*. [28] and Kumar *et al*. [6]. More recently, a structural genomics based study carried out by Chakravarty and Varadarajan [29] reported that leucine is preferentially substituted by the β-branched residues valine and isoleucine, at buried sites.

Several studies have demonstrated in the past that leucine has a slightly higher α-helix propensity than isoleucine and, generally, β-branched residues [27,30]. This assumption, which is apparently in contrast with the results obtained by this work, derives from substitution experiments in short polyalanine α-helices-forming peptides in water [31]. This process is mainly associated with the loss of conformational entropy of residues during the folding of α-helices in an aqueous environment: freezing side chain with fewer internal rotational degrees in the α-helix conformation would be entropically less expensive. However, it must be noted that these experiments, and many derived propensity scales, do not take into account solvent entropy effects. As discussed by Creamer and Rose [30], neglect of solvent entropy appears justified for a peptide side chain because no significant differences in solvation energy are expected in the side chain of a solitary polyalanyl helix during a helix-coil transition. In either case, the side chain is highly solvent-exposed. The same situation would not be appropriate for a protein helix that, upon association with the remainder of the molecule, engages a solvent-shielded interaction surface. In this study, only the α-helices composing the SCRs and therefore mostly found in the protein core were considered for further investigation. Therefore, application of helix propensity scales might be not appropriate in this case. For example, Li and Deber [15] have shown that α-helices propensity scales are not appropriate for non aqueous environments and that β-branched amino acids, as valine and isoleucine, rank among the best helix promoters in an apolar environment, as a lipid bilayer.

On the other side, hydrophobic contacts deriving by side chain interactions could play a role of great importance in the stabilization of the α-helices composing the SCRs of hyperthermostable proteins. At temperatures above 80°C, the hydrophobic effect, that is considered to be a dominant force in protein folding [32,33], is mainly enthalpy driven [34]. In fact, while at high temperatures the entropy contribution to the protein stability tends to zero, the loss or gain of van der Waals interactions acquires increased importance. For example, constructing 15 Barnase mutants in which hydrophobic interactions were deleted, Serrano *et al*. [35] found a strong correlation between the degree of Barnase destabilization and the number of methyl side chain groups that were lost (*r *= 0.91). These data agree with the preferred substitutions (*R*_Ala→Val _= 3.20; *R*_Val→Ile _= 6.31) observed in the CHCs of our datasets.

## Conclusion

In conclusion, taken together the obtained results indicate the preference, in the hydrophobic contacts, for isoleucine and valine residues as an important feature contributing to the enhanced thermostability of α-helices in hyperthermophilic proteins, possibly occurring through a decreased flexibility of these elements of secondary structure. This effect, in turn, may be due to an increased number of buried methyl groups in protein core and/or a better packing of α-helices with the rest of the structure, caused by the presence of hydrophobic β-branched side chains.

Despite the advances in the design of hyperthermostable protein variants [17], a potential drawback of these approaches is still constituted by the time consumed by computer algorithms for exploring the whole sequence protein space. Other things being equal, focussing on the apolar contact area of the α-helices of the protein core through substitutions increasing the number of methyl side chain groups and/or resulting in a better packing of the secondary structure elements, will potentially give clues for the thermostabilization of the protein.

## Methods

### Data Collection

Hyperthermophilic and thermophilic protein structures were retrieved from Protein Data Bank (PDB)[36], by initially searching for the words "thermo", "thermophile" and "hyperthermophile". This search yielded about 300 proteins and their corresponding sources. An additional search was then performed using as query the name of such organisms, after having assessed that their optimal growth temperatures were between 50°C and 80°C for thermophiles, and above 80°C for hyperthermophiles [3]. Optimal growth temperatures for each organism were obtained from *Entrez *[37] and the "Prokaryotic Growth Temperature Database" [38]. As a first refinement step, the entries in which protein structures were determined by nuclear magnetic resonance (NMR) were discarded, yielding about 1563 crystal structures.

As a second refinement step, all the entries were examined by means of the PISCES tool [39], and culled from the original dataset by maximum percentage of identity (90%), maximum resolution (2.0 Å), maximum *R-value *(0.25) and minimum chain length (50 residues) criteria. Furthermore, a second dataset was collected following less stringent criteria (maximum resolution at 3.0 Å and maximum *R-value *at 0.30), in order to cull a greater number of structures. This second step yielded 458 and 767 proteins for dataset *A *and *B*, respectively. Each dataset was then further reduced by eliminating proteins displaying any structural defect, such as missing side-chains or chain breaks due to missing residues, using the MAXIT tool, available at [46]. At the end of this refinement step, 93 and 144 structures comprised dataset *A *and *B*, respectively.

Each structure of the two datasets was then exploited to check for the presence in PDB of a mesophilic counterpart. To this purpose, a search with the blast tool [40,41] was carried out, adopting the following criteria: 30% minimum sequence identity, that is usually accepted as a threshold value to assure a homology relationship between two proteins [42]; 90% maximum sequence identity, in order to avoid any redundancy of data; 40% maximum difference in length between the sequences, to avoid the presence of large indels between the two structures. Furthermore, the retrieved mesophilic proteins had to satisfy the same above described structural criteria to be accepted. In those cases yielding several mesophilic homologous structures available for each hyperthermophic/mesophilic protein, the one displaying the highest percent of sequence identity was collected. At the end of this search, 38 protein pairs for dataset *A *(14 thermophilic/mesophilic pairs and 24 hyperthermophilic/mesophilic pairs) and 59 protein pairs for dataset *B *(22 thermophilic/mesophilic pairs and 37 hyperthermophilic/mesophilic pairs) were collected (Table 1 and Table 2).

### Computation of the Apolar Contact Area

Computation of the total apolar contact area between the residues of each structure composing dataset *A *and *B *was carried out by means of the pdb_np_cont tool [43], which computes pairwise atom contact areas between non-polar atoms from structural protein data in a standard PDB coordinate file. Briefly, this method is based on the classification of points located on a sphere of interaction radius, surrounding each non-polar atom. The interaction radius is the van der Waals radius of each atom type, plus the radius of a water molecule. The output of this program was utilized to calculate the pairwise residue contact areas for every possible pair of residues belonging to the structures analysed. Heteroatoms were ignored. The total apolar contact area was then normalized by sequence length of each protein structure.

In order to assess the role played by the hydrophobic contacts in the stabilization of the protein core, at high temperatures, each pair of homologous hyperthermophilic/mesophilic and thermophilic/mesophilic structures was initially superposed by means of the CE-MC tool [44]. The resulting alignment was then utilized to derive manually refined structural alignments. Every pair of structures was visually inspected and, where necessary, modified to optimise the matching of several structural features, including observed secondary elements, functionally conserved residues and hydrophobic regions, in order to give the most accurate structural alignment.

Each structural alignment obtained as described above was utilized to identify the common core and the structurally conserved regions between the pairs of proteins taken into consideration (SCRs). SCRs were defined as regions displaying a similar local conformation, with a mean positional RMSD of the equivalent α-carbon positions of the structures superposed ≤ 3.0 Å [18], lacking indels (insertions and deletions) and composed of at least three consecutive residues. For every structurally equivalent position of the pairwise structural alignment, the RMSD from the center of mass of the structurally equivalent C_α _atoms was computed. To avoid the presence of SCRs with indels, positions with gaps were not considered. A window of size *w *= 3 positions was then scrolled through the alignment and used to define seed positions with a mean RMSD ≤ 3.0 Å. Each time a seed position was found, *w *was increased iteratively by one position until the mean score remained belove 3.0 Å, or until the window reached the end of the alignment. The obtained SCRs were then visually inspected to avoid the possible presence of regions with different conformations. Then, the hydrophobic contacts involving pairs of topologically equivalent residues in both of the structures analysed (Conserved Hydrophobic Contacts, CHCs) were extracted from the identified SCRs. The SCR_FIND and CHC_FIND tools [19] were utilized to this purpose.

The differences observed in the amount of apolar contact area between the SCRs of the hyperthermophilic/mesophilic and thermophilic/mesophilic protein pairs were further investigated through the analysis of such differences in the regular secondary structure elements: α-helices and β-strands. Secondary structures were determined by using the program DSSP [45].

The amount of apolar contact area measured in the SCRs and secondary structure elements of each structure were finally normalized by the number of residues belonging to SCRs, α-helices and β-strands, respectively.

### Amino acid Composition of the residues involved in CHCs

Differences in amino acid composition were measured by:

$$D^{aa}=\frac{n^{T}(aa)}{n_{T}^{aa}}-\frac{n^{M}(aa)}{n_{M}^{aa}}$$

where *D*^*aa *^is the difference in amino acid composition for residue *aa*, *n*^*T *^and *n*^*M *^are the number of residues of type *aa *in hyperthermophilic/thermophilic (*T*) and mesophilic (*M*) structures and *n*^*aa *^is the total number of residues in hyperthermophilic, thermophilic (*T*) and mesophilic (*M*) structures.

The *D*^*aa *^values measured for each pair of the structures analysed were then used to calculate the difference in amino acid composition *C*^*aa *^over the *k *pairs composing dataset *A *and dataset *B*:

$$C^{aa}=\sum_{k}D^{aa}$$

The mean and standard deviation for the *C*^*aa *^elements were determined; the significance *R*^*aa *^of the difference in amino acid composition for residue *aa *was then calculated by dividing the difference between *C*^*aa *^and the overall mean $\bar{C}$ by the standard deviation σ:

$$R^{aa}=\frac{\left|C^{aa}-\bar{C}\right|}{\sigma }$$

*R*^*aa *^values ≥ 3.0 standard deviations (corresponding to a probability *P *≤ 0.01 that the observed difference was obtained by chance) from the mean value were considered statistically significant.

### Preferred amino acid pairs in CHCs

Preferred amino acid pairs forming hydrophobic contacts were identified by computing the number of times a particular pair of residues comprised in SCRs makes a hydrophobic contact. The obtained counts were then normalized by the number of pairs of interacting residues present in the SCRs of the structure taken into consideration. An interaction matrix reporting the differences in the number of apolar contacts for each possible pair of residues, between hyperthermophilic/mesophilic and thermophilic/mesophilic structures, was derived:

$$C^{XY}=\sum_{k}{\left(C_{T}^{XY}-C_{M}^{XY}\right)}_{k}$$

where *k *represents the number of elements of dataset *A *or *B*, *C*^*XY *^is the element of the matrix reporting the differences in the number of apolar contacts for the pair *XY *of interacting residues, *C*_*T *_and *C*_*M *_are the normalized counts for the hyperthermophilic/thermophilic and the mesophilic proteins, respectively.

The mean and standard deviation for the non-zero elements of the overall interaction matrix were determined; the significance *R*^*XY *^of the interaction *XY *was then calculated by dividing the difference between *C*^*XY *^and the overall matrix mean $\bar{C}$ by the standard deviation σ:

$$R^{XY}=\frac{\left|C^{XY}-\bar{C}\right|}{\sigma }$$

*R*^*XY *^values ≥ 3.0 standard deviations (corresponding to a probability *P *≤ 0.01 that the observed difference was obtained by chance) from the mean value were considered statistically significant.

### Preferred amino acid substitutions in CHCs

Amino acid substitutions of residues involved in the formation of conserved hydrophobic contacts between hyperthermophilic and mesophilic proteins were determined by analysing the alignment of the SCRs of each pair. For each residue *X*, belonging to a mesophilic protein and involved in making CHCs, *aa*_X→Y _was defined as the number of times *X *is substituted by the residue *Y *of the hyperthermophilic sequence. Likewise, *aa*_Y→X _is defined. Therefore, a substitution matrix can be obtained by computing the difference between *aa*_X→Y _and *aa*_Y→X _over the whole dataset of protein pairs *k*, according to:

$$C^{S}=\sum_{k}\left(\sum aa_{X\to Y}-\sum aa_{Y\to X}\right)$$

where C^S ^is the element of the substitution matrix.

The mean and standard deviation for the non-zero elements of the overall exchange matrix were determined; the significance *R*_*XY *_of the exchange *X → Y *was then calculated by dividing the difference between *C*^*S*^, and the overall matrix mean $\bar{C}$ by the standard deviation σ:

$$R_{XY}=\frac{C^{S}-\bar{C}}{\sigma }$$

*R*_*XY *_values ≥ 3.0 standard deviations (corresponding to a probability *P *≤ 0.01 that the observed difference was obtained by chance) from the mean value were considered statistically significant.

### Statistical significance

The statistical significance of the observed differences of *ACA *between hyper/thermophilic proteins and their mesophilic counterparts was assessed with a paired *t*-test (applied to every pair of structures composing dataset A and dataset B, respectively), to judge the rejection of the null hypothesis (*t *> 2.0; P(*t*) < 5%). The null hypothesis to be rejected with the paired *t*-test analysis is that there is not a significant difference between the measured values of ACA in the hyper/thermophilic and mesophilic proteins.

In order to ensure that the measured *P(t) *was not biased by the extreme values of the distributions, the *t*-test validation analyses were repeated, removing the highest and lowest values from the datasets.

The Shapiro-Wilk normality test was applied to judge the distribution of the obtained values for the two datasets. The null hypothesis of this test is that the analysed samples of data are taken from a Gaussian distribution; therefore, the returned *P(t) *of this test represents a criteria of acceptance or rejection of the null hypothesis. A *P(t) *< 0.05 was considered statistically significant to reject the supposition of normality.

## Authors' contributions

AP conceived the study, interpreted the data and wrote the final manuscript. AP and RS both contributed source code. RS collected the structures, the datasets and implemented most of the various computational analyses. SP supervised the study and helped draft the manuscript. FB coordinated the study and helped to draft the manuscript. All authors read and approved the final manuscript.

## Supplementary Material

Additional file 1 **Paired T-Test analysis, datasets and distribution of data**. This file includes the datasets A and B, described in this paper, and the statistical analysis of the distribution of *ACA*. Click here for file
